# Health Benefits of Vegetarian Diets: An Insight into the Main Topics

**DOI:** 10.3390/foods13152398

**Published:** 2024-07-29

**Authors:** Luciana Baroni, Gianluca Rizzo, Alexey Vladimirovich Galchenko, Martina Zavoli, Luca Serventi, Maurizio Battino

**Affiliations:** 1 Scientific Society for Vegetarian Nutrition—SSNV, 30171 Venice, Italy; luciana.baroni@scienzavegetariana.it (L.B.); alexey.galchenko@scienzavegetariana.it (A.V.G.); martina.zavoli@scienzavegetariana.it (M.Z.); 2 Earth Philosophical Society “Melodia Vitae”, International, Toronto, CA M9A4X9, Canada; 3 The New Zealand Institute for Plant and Food Research Limited, Lincoln 7608, New Zealand; luca.serventi@plantandfood.co.nz; 4 Joint Laboratory on Food Science, Nutrition, and Intelligent Processing of Foods, Polytechnic University of Marche, Italy, Universidad Europea del Atlántico Spain and Jiangsu University, China, Via Pietro Ranieri 65, 60131 Ancona, Italy; m.a.battino@univpm.it; 5 International Joint Research Laboratory of Intelligent Agriculture and Agri-Products Processing, Jiangsu University, Zhenjiang 212013, China; 6 Department of Clinical Sciences, Polytechnic University of Marche, Via Pietro Ranieri 65, 60131 Ancona, Italy; 7 Research Group on Foods, Nutritional Biochemistry and Health, Universidad Europea del Atlántico, Isabel Torres 21, 39011 Santander, Spain

**Keywords:** vegetarian diet, lacto-ovo-vegetarian diet, vegan diet, plant-based diet, health, noncommunicable diseases

## Abstract

Vegetarian diets are plant-based diets including all the edible foods from the Plant Kingdom, such as grains, legumes, vegetables, fruits, nuts, and seeds. Dairy and eggs can be added in small amounts in the lacto-ovo-vegetarian subtype, or not at all in the vegan subtype. The abundance of non-processed plant foods—typical of all well-planned diets, including vegetarian ones—can provide the body with numerous protective factors (fiber, phytocompounds), while limiting the intake of harmful nutrients like saturated fats, heme-iron, and cholesterol. The beneficial effects on health of this balance have been reported for many main chronic diseases, in both observational and intervention studies. The scientific literature indicates that vegetarians have a lower risk of certain types of cancer, overall cancer, overweight-obesity, type 2 diabetes, dyslipidemia, hypertension, and vascular diseases. Since the trend of following a vegetarian diet is increasing among citizens of developed countries, the knowledge in the field will benefit from further studies confirming the consistency of these findings and clarifying the effects of vegetarian diets on other controversial topics.

## 1. Introduction

**Figure 1:**
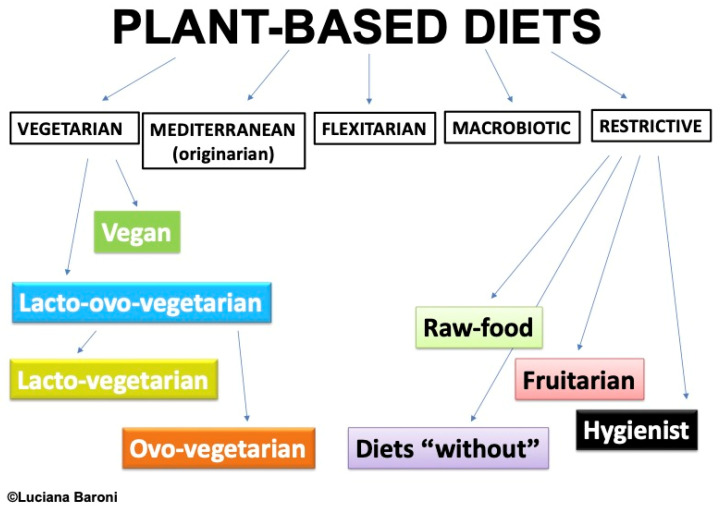
Different types of plant-based diets: a proposed classification.

Plant-based diets are mainly, or exclusively, based on plant foods, which include all the edible foods from the Plant Kingdom. So, plant-based diets are based on grains, legumes, fruits, vegetables, nuts, and seeds. The unique characteristics of plant foods (obviously in their non-processed form) include being the exclusive source of fiber and phytocompounds, whose multiple beneficial effects on our health have been shown previously [1]. To this large basis of plant foods, small amounts of animal foods, both indirect (i.e., milk and eggs, such as in the lacto-ovo-vegetarian diet), and direct (i.e., any kind of flesh, such as in the original Mediterranean diet) can be added, or not. This means that vegetarian diets are a subtype of plant-based diet that includes all the foods from the five plant food groups and excludes any kind of flesh (in lacto-ovo-vegetarian, LOV), and also milk and eggs (in vegan, VN). Such foods, however, can be minimally included in other plant-based diets (e.g., flexitarian, Mediterranean) [2] (Figure 1).

The composition of plant-based diets in developed countries differs from that in developing ones. In the latter, they may be an “obligate” choice due to food scarcity, resulting in diets that can be monotone and nutritionally inadequate. Conversely, in developed countries, food availability is not a problem, allowing people to choose plant-based diets for ethical, health, and, above all, ecological reasons [3], making this trend an increasing one [4,5,6].

It has been reported that, around the world, about 75 million people voluntarily choose to follow a vegetarian diet, while 1450 million are forced to follow this kind of diet by socio-economic reasons [7].

The nutrient adequacy of vegetarian diets (both for LOV and VN) has been confirmed by leading nutrition experts (e.g., the Academy of Nutrition and Dietetics—AND, the Dietitians of Canada—DoC, the German Nutrition Society—DGE, and the Italian Society for Human Nutrition—SINU) [8,9,10,11,12]. Characteristics of a vegetarian diet associated with its nutritional adequacy include consuming a variety of plant foods (mainly non-processed) from all the food groups, in amounts necessary to meet the calorie requirements, and paying attention to the critical nutrients of the diet, i.e., vitamin B12, vitamin D, calcium, and omega-3 fatty acids [13].

An Optimal Diet is a diet that promotes health and longevity [14], and is typical of populations with a reduced incidence of chronic disease. The Optimal Diet is a plant-based diet, abounding in complex carbohydrates, fiber, and water, while limiting fats, cholesterol, salt, and sugars. In a plant-based diet, complex carbohydrates are mainly provided by grains and legumes, fiber by all non-processed plant foods, and water is the main component of vegetables and fruits. Protein is found in the protein-rich plant foods (legumes) but is also present in lower amounts in other plant foods (grains, vegetables, nuts and seeds). Varied consumption of foods from all the dietary groups (grains, legumes, fruits, vegetables, nuts, and seeds) ensures dietary adequacy for protein, which in the Optimal Diet should represent no more than 10–15% of total energy. Therefore, a vegetarian diet is very similar to an Optimal Diet and is expected to benefit health outcomes [15].

The aim of this review is to highlight the main benefits of vegetarian diets on the risk and course of the most common non-communicable chronic diseases—the leading cause of death in developed countries. 

Only meta-analyses of observational and intervention studies on vegetarian (lacto-ovo-vegetarian and vegan) dietary patterns have been included, without any restriction for age, gender, ethnicity, geographical origin, or socio-economic status. All the other plant-based dietary patterns have been excluded (see Figure 1). We discuss only the topics in which two or more meta-analyses were available.

According to the previously set inclusion criteria, we found two or more meta-analyses about cancer, overweight-obesity, type 2 diabetes, dyslipidemia, hypertension, and vascular disease. We included in the review also metabolic syndrome, since the single factors composing it had been discussed in the previous subsections. All the health effects for these outcomes were favorable or non-significant. We found a meta-analysis on bone health [16] with uncertain unfavorable effects, but it was unique, which led us to exclude bone health from the review.

The identified meta-analyses have been conducted on subjects participating in observational studies, healthy at the enrollment, or on subjects affected by cardiometabolic conditions and cancer, who participated in intervention studies with a vegetarian diet, whose effect was compared to the effect of the habitual diet or diets for weight loss or diabetic management.

## 2. Health Effects

**Figure 2:**
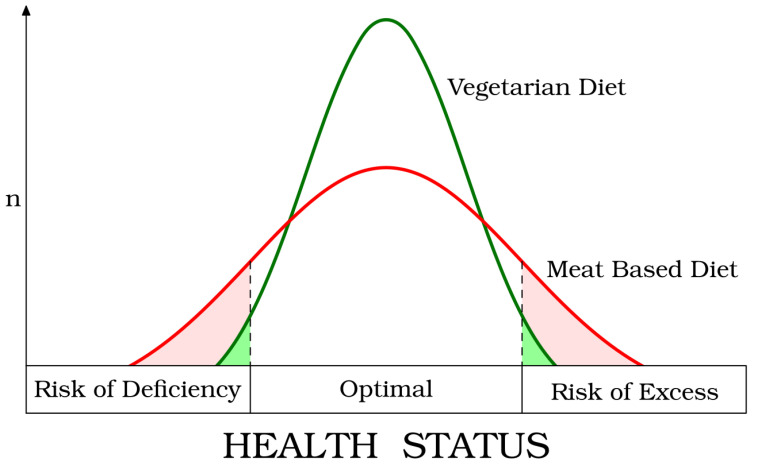
The “Paradigm Shift” proposed by Joan Sabaté (reproduced with the permission of Prof. Sabaté).

Evidence-based research has attributed beneficial health effects to plant-based diets. As early as 2003, Prof. Sabaté proposed a “paradigm shift” to suggest why more people following a vegetarian diet could achieve optimal health. He suggested revising the concepts of “risk of dietary excess” and of “risk of dietary deficiency”. The former should classify the intake of harmful nutrients or foods (e.g., saturated fats, heme-iron, meat), characteristic of meat-based diets. Conversely, the harmful “risk of dietary deficiency” should contemplate the low intake of beneficial nutrients and foods, such as all the unprocessed plant foods, fiber, and phytocompounds with antioxidant and anti-inflammatory effects [1,17,18]. Since a vegetarian diet is mainly based on plant foods, the risk of deficiency of protective factors and the risk of excess of harmful factors is reduced, explaining the better health of vegetarians (Figure 2) [19].

This means that the health effect of nutritionally adequate plant-based diets can be commonly attributed to the higher intake of beneficial substances rather than the mere elimination of meat [20]. Plant foods and their components can synergically act to reduce the risk of the more common chronic non-communicable diseases (vascular diseases, type 2 diabetes, overweight-obesity, hypertension, certain types and total cancer).

Some common mechanisms beneficial on health, elicited by plant foods, can act through synergic mechanisms, which include the composition of the microbiota and the mitigation of inflammation and oxidative stress.

### 2.1. Gut Microbiota

It is now well established that the gut microbiota influences human health and brain functions, and that diet can modulate the gut microbiota’s composition [21,22]. In particular, plant foods can promote a diverse ecosystem of beneficial bacteria; in comparison to omnivores, vegetarians have greater richness and diversity in their fecal microbiota. A higher *Prevotella*/*Bacteroides* ratio and a lower Firmicutes/Bacteroidetes ratio have been reported in vegetarians [23]. The bacterial fermentation of dietary fiber in the gut promotes the production of short-chain fatty acids (SCFAs, e.g., acetate, propionate, and butyrate), and elicits beneficial effects on immunity, inflammation, lipid and glucose metabolism, gut barrier and blood–brain barrier integrity [24,25,26]. Moreover, plant-based diets have been reported to have beneficial effects on plasma and urinary TMAO concentration (trimethylamine N-oxide, a recognized indicator of cardiovascular disease risk). Increased TMAO production is associated with diets high in protein, particularly of animal origin, while, conversely, plant-based diets were shown to reduce TMAO levels effectively [27].

### 2.2. Chronic Inflammation

Low-grade chronic inflammation, mediated by macrophages in major tissues [28], represents a common underlying factor in chronic diseases [29,30,31,32,33]. It has been linked to gut microbiota, obesity, and diet, and can also be caused by a metabolic imbalance (meta-inflammation) [34,35,36]. A clinical trial performed on CAD (coronary artery disease) patients, the EVADE CAD trial, compared the effect of the American Heart Association diet with the effect of a VN diet on 100 randomized participants. After 8 weeks, a −32% significant reduction in hsCRP (high-sensitivity C-Reactive Protein, a marker of risk for major adverse cardiovascular outcomes in CAD) was observed in VNs [37]. Some systematic reviews and meta-analyses supported the presence of lower levels of inflammatory biomarkers, mainly hsCRP, in vegetarians [38,39,40,41], suggesting that plant-based diets could reduce the risk of chronic diseases even by acting on circulating levels of these biomarkers.

### 2.3. Oxidative Stress

The damage to cells and tissues caused by free radicals (oxidative stress) results from the imbalance between the production and accumulation of reactive oxygen species (ROS, by-products of oxygen metabolism) and the body’s ability to neutralize these reactive products [42]. Compounds with antioxidant activity—bio compounds like phenolic acids, flavonoids, organic sulfides, and carotenoids, vitamins C and E—are naturally contained in plants, especially when grown in harsh environments [43]. Their ability to protect the body from the damage caused by oxidative stress is well explored and recognized; the consumption of natural plant foods, rich in antioxidants, has been associated with a reduction in the risk of major chronic diseases [44]. Even though oxidative stress can be caused by chemical environmental contaminants, iron accumulation in tissues can also generate oxidative stress [45,46,47,48]. Research has shown that not only are iron stores lower in vegetarians compared to non-vegetarians [49], but a vegetarian diet can also improve the levels of oxidative stress markers and the oxidant–antioxidant balance compared to an omnivore diet [50,51].

### 2.4. Energy Metabolism

Diets rich in plant foods, like vegetarian diets, have a low-calorie density, mitigating the risk of energy intake excess. In a clinical trial that compared the effect of a low-fat vegan diet with the habitual diet, providing an ad libitum intake of food, the energy intake was reduced of about 500 kcal/d in the intervention group [52]. Moreover, it has been suggested that the thermic effect of foods increased after the consumption of large intakes of carbohydrates and low intakes of fats [53]. A clinical trial performed with a low-fat vegan diet resulted in a reduced energy intake, increased postprandial metabolism and insulin sensitivity, in comparison to a habitual diet [52]. The same advantage was reproduced also when comparing a low-fat vegan diet with another plant-based diet, the Mediterranean one [54], where energy intake, body weight, blood lipid levels, and insulin sensitivity significantly improved in the intervention group. Finally, plant foods have been shown to positively affect gastro-intestinal hormones in comparison to animal foods [55,56], favorably influencing the regulation of appetite and the intake of food and energy.

## 3. Beneficial Health Outcomes

Research on the health effects of plant-based diets began in the past century, and has shown a growing trend, allowing reliance on solid evidence-based data. The Umbrella Review by Oussalah [57] found that “*in comparison to omnivorous diets, vegetarian diets were associated with a reduced risk of negative health outcomes with a pooled ES (Effect Size) of 0.886 (p < 0.001)*”. As is often the case in nutrition research, some data can be controversial, since nutrition epidemiology is not an exact science. Nevertheless, strong convergence in the results of many studies is evidenced by the numerous meta-analyses published in the last decade, which are summarized in this section.

### 3.1. Cancer

Cancer is a multifactorial disease, resulting from the interaction between genetic and environmental factors, causing DNA damage that perturbs cell growth and function, allowing it to proliferate uncontrollably. Consequently, cancer can invade contiguous tissues and metastasize to other body sites typical of each cancer type. 

It was established early on that diet could play a crucial role in both the onset and progression of many types of cancer. As early as 1981, Doll and Peto estimated that approximately 35% of cancer deaths in the United States were potentially avoidable by modifying diet, with a variability ranging from 10% to 70%, depending on the type of cancer [58]. In 1990, Willett confirmed this estimate, suggesting that 32% of cancer may be avoidable trough dietary changes, even if he considered a narrower variation range (20–42%) [59]. The mechanisms involved are multiple and complex, but dietary risk factors can act mainly through genotoxic, cytotoxic, and inflammatory mechanisms [60]. Red meat, both unprocessed and processed, represents a well-recognized risk factor, while the risk related to other foods has not been unequivocally determined yet. Nevertheless, the WCRF Report recognizes the protective effect of plant foods, such as whole grains, non-starchy vegetables, fruits, unprocessed plant foods containing fiber and beta-carotene, and soy foods [61].

Observational studies have evaluated the association between diet and cancer risk in large cohorts. In the Oxford cohort, the 12-year follow-up of more than 61,000 subjects allowed to identify a risk reduction in gastric cancer (−64%), bladder cancer (−53%), lympho-hemopoietic cancers (−45%) in vegetarians compared to non-vegetarians. The risk for total cancer was also reduced in fish-eaters (−18%) and vegetarians (−12%) [62]. A study performed on the Adventist cohort in 2013, including about 70,000 subjects over a follow-up of about 4 years, found risk reductions for cancers in LOVs of −25% for gastrointestinal cancers, and in VNs of −34% for female cancers and −16% for all cancers [63]. A more recent study on more than 470,000 subjects recruited through the UK Biobank, with a follow-up of about 11 years, showed a reduced risk of cancer in vegetarians for colon-rectum (−22%), post-menopausal breast (−18%), prostate (−31%), and for all cancers (−14%) [64].

Meta-analyses of observational studies, overall, suggest a reduction in all-cancer risk among vegetarians. LOVs and VNs have been recruited under the “vegetarian” group, though some reports separated the two subtypes of diets. Huang collected seven studies (about 125,000 subjects) and found a risk reduction of −18% for all cancers in vegetarians [65]. Dinu et al. distinguished LOVs from VNs (respectively, 38,033 and 7168 participants) reporting a risk reduction for all cancers of −8% in LOVs and −15% in VNs [66]. Parra-Soto et al. reported a risk reduction for all cancers of −10% and for colon-rectum cancer of −15% in vegetarians, in comparison to meat-eaters, in a sample of about 550,000 subjects [67]. Zhao et al. evaluated the relationship between plant-based diet and the risk of digestive system cancers at different sites on 49 studies and more than 3 million subjects. The risk reduction was statistically significant for all types of cancer of the digestive system and for specific sites such as pancreas, colon-rectum, colon, rectum, both in cohort and case-control studies (−18% and −30% for all digestive system cancers, −29% and −35% for pancreatic cancer, −24% and −33% for colorectal cancer, −12% and −7% for colon cancer, −16% and −9% for rectal cancer, −39% and −39% for liver cancer, respectively). A significant reduction was also present for gastric cancer (−42%), esophageal cancer (−38%), and pharyngolaryngeal cancer (−36%) only in case-control studies. A further sub-analysis distinguishing the different plant-based dietary patterns identified a reduction in the risk for all cancers of −20% in cohort studies and −38% in case-control studies in VN diets, and −18% in cohort studies and −28% in case-control studies for the other plant-based patterns [68].

In a 2-year intervention study on subjects affected by prostate cancer undergoing an intensive lifestyle program that included a vegan low-fat diet, only 5% of patients required traditional treatment in the intervention group, compared to 27% in the control group [69].

### 3.2. Overweight-Obesity

Certainly, overweight-obesity (OO) represents, amongst all, the only “well recognizable” chronic disease. It is widespread in developed countries, affecting lower economic classes of citizens; on the contrary, in developing countries, OO is a marker of wealth. OO is not only a disease in itself, but it represents also a risk factor for many other pathological conditions, such as diabetes and cancer. OO results from an unbalance of energy management, which can derive from a reduction in movement-related calorie expenditure and/or an excess of dietary calorie consumption, the latter linked to the quality of the diet.

An increased amount of animal foods in the diet seems to promote an increase in Body Weight (BW). In a study conducted on the Adventist cohort in 2009 by Tonstad et al., a clear increasing trend in Body Mass Index (BMI, kg/m^2^) was evident from VNs to meat-eaters. The shift from meat-eater pattern towards semi-vegetarian, fish-eater, LOV, and VN patterns showed a progressively decreasing BMI, with a difference between meat-eaters and VNs of 5.2 kg/m^2^ [70]. A plant-food diet can be favorable not only in preventing OO, but also for its non-pharmacological treatment. In a two-year intervention study by Turner-McGrievy et al., the effect of a VN diet was compared with the effect of the NCEP (National Cholesterol Education Program) diet on 62 women. After the first year, BW decreased in both groups but, during the second year, the control group reversed the trend, while the intervention group maintained and improved the BW loss [71].

Various meta-analyses of studies evaluating the association of different dietary patterns with BW/BMI consistently show that plant-based patterns elicit a beneficial effect, both in preventing and treating the disease [66,72,73,74,75,76]. The results are summarized in the following Table 1. Worth noting is the analysis by Benatar et al., which distinguished the total sample and two subgroups: Asian and non-Asian. Data in the table refer to the whole sample and the subgroups: only in non-Asian subjects was the BMI reduction statistically significant (−1.92 vs. −0.2 of Asians) [73]. We describe the results of this meta-analysis on other health outcomes in the following paragraphs. For all the outcomes, the authors attributed this finding to differences in the quality and composition of the diet in the two subgroups: vegans included in the studies from Taiwan may adhere less strictly to a vegan diet, and the dietary pattern for omnivores in Asia may include fewer animal products than for non-Asian countries, so the differences between omnivores and vegans may be less pronounced. The authors concluded also that, based on these observations, the subgroup analysis excluding Asian studies may provide a more reliable estimate of the effects of a strictly vegan diet compared to an omnivorous diet in non-Asian countries.

On this specific topic, the AND position paper recommended that professionals be aware of the evidence supporting the use of vegetarian diets for achieving and maintaining a healthy weight, and also of the therapeutic use of vegetarian diets for treating OO, compared to alternative omnivorous diets for weight loss [8]. Additionally, the Italian Society for Human Nutrition (SINU) reports that observational studies showed advantages of vegetarian diets over non-vegetarian diets in terms of OO risk, and in intervention studies the use of vegetarian diets, mainly low-fat vegan ones, led to greater weight loss than non-vegetarian diets [77].

### 3.3. Diabetes (Type 2)

One of the main complications of OO is type 2 diabetes (T2D), an alteration of glucose metabolism which can evolve with acute and chronic complications and increase cardiovascular risk. It has been extensively reported that meat consumption increases T2D risk. In a 2011 meta-analysis including about 440,000 subjects, a 100 g daily increase in red meat consumption increased T2D risk by 19%, and a 50 g daily increase in processed meat consumption increased T2D risk by 51% [78]. It has been suggested that meat may act through multiple mechanisms, encompassing its content in heme-iron, saturated fatty acids, branched amino acids, choline/carnitine, nitrosamines and nitrose compounds, AGEs (Advanced Glycation End-products), and eliciting insulin resistance. Conversely, plant foods have been reported as protective against insulin resistance and T2D, mainly due to their fiber content [79]. In a clinical trial, a low-fat vegan diet, promoting ad libitum intake of non-processed plant foods and the avoidance of added fat, decreased dietary AGE intake by −73%, compared to no change in the Mediterranean diet. Changes in dietary AGEs correlated with changes in BW, which decreased by 6 kg, compared with no change in the Mediterranean diet [80].

In the Adventist cohort, the prevalence of diabetes among about 61,000 participants at enrollment was higher in diets including animal foods, and the lowest in VN participants (7.6% in non-vegetarians and 2.9% in VNs) [70]. In the same cohort, after a 2-year follow-up of about 41,000 non-diabetic subjects at enrollment, the risk of developing diabetes, compared to non-vegetarians, was reduced by −62% in VNs, −51% in semi-vegetarians and −38% in LOVs [81]. The meta-analysis by Lee and Park found that vegetarian diets, compared to non-vegetarian diets, are associated with a −27% reduction in T2D risk [82].

A plant-based diet has also shown effectiveness in the non-pharmacological treatment of hyperglycemia. In a multicenter clinical trial, a vegan low-fat diet reduced HbA1c levels by −0.74% after 18 weeks, compared to the habitual diet [83]. Kahleova et al. found that this kind of diet is effective in reducing hepatocellular and intramyocellular fat and increasing insulin sensitivity [52].

The results of the various meta-analyses [66,73,75,84,85] are consistent with the recommendation of the Canadian Diabetes Association for practitioners, regarding the use of a plant-based diet in the management of diabetes [86]. A plant-based diet, particularly if vegan low-fat, is an effective weapon to prevent and treat the disease. Table 2 summarizes the results of the meta-analyses. Also in this case, Benatar et al. subdivided the sample, and found that non-Asian population had the most significant advantages (−0.39 mmol/L vs. −0.12 mmol/L for Asians) [73].

According to the AND position paper, vegetarian diets, characterized by micronutrient-dense, high-fiber plant foods, are effective in the prevention and in the treatment of T2D. This is because the typical reduced intake of saturated fat and the high consumption of vegetables, fruits, whole grains, legumes, soy products, nuts, and seeds (all rich in fiber and phytochemicals) can improve serum glucose control [8]. The comprehensive review by SINU reports that both cohort and intervention studies have shown that vegetarian diets have positive effects on T2D risk and management [77].

### 3.4. Dyslipidemia

Total cholesterol (TC), LDL-cholesterol (LDL-C), and non-HDL cholesterol (non-HDL-C) levels represent major cardiovascular risk factors. Cholesterol levels are affected, amongst other factors (smoke, inactivity, genetics, sex, age), by diet and the presence of other diseases like OO and T2D. Dietary factors that negatively influence blood cholesterol levels are saturated fats, trans fats, and cholesterol, mainly found in animal foods and some processed plant foods. Triglyceride blood level increases can be caused by alcohol and sugar consumption. In the Oxford cohort, at enrollment, participants’ total cholesterol (TC) and apoB-lipoprotein levels were highest in meat-eaters and lowest in VNs, for both sexes [87]. In the GEICO study, a multicenter intervention trial, a reduction in TC levels by −13.7 mg/dL after 18 weeks of an ad libitum low-fat vegan diet was observed, compared to controls on a habitual diet [83].

A meta-analysis of 30 observational studies (more than 10,000 participants) shows that vegetarian diets are associated with a reduction in blood TC, LDL-C, and HDL-C levels [88]. Results of the other two meta-analyses reported in Table 3 [66,73] support that, when LOVs and VNs are analyzed separately, VN diets tend to be more effective than LOV ones in controlling cholesterol blood levels. Among the various authors, only Benatar et al. found a significant reduction in triglyceride blood levels in VNs, compared to omnivores (−12.4 mg/dL in the total sample) [73].

Meta-analyses of intervention studies also support the effectiveness of vegetarian diets in reducing cholesterol blood levels [68,69,79,82] as shown in Table 4.

Finally, we should comment on the HDL-C controversy, as some meta-analyses found a reduction, sometimes statistically significant, in HDL-C blood levels in vegetarian diets. This finding is intriguing, because it has been suggested that HDL-C may represent a protective factor against cardiovascular disease. Nevertheless, the meta-analysis of randomized controlled trials performed by Keene et al., including 117,411 patients, has shown that an increase in HDL-C levels does not decrease vascular risk [89]. Moreover, genetically determined low HDL-C levels are not associated with an increased risk for ischemic heart disease [90]. Other authors report that “*HDL-C level is unlikely to represent a CV-specific risk factor given similarities in its associations with non-CV outcomes*.” [91].

In conclusion, the two main position papers on vegetarian diets specifically addressed blood lipids. According to the AND position paper: “*Low intake of saturated fat and high intakes of vegetables, fruits, whole grains, legumes, soy products, nuts, and seeds (all rich in fiber and phytochemicals) are characteristics of vegetarian and vegan diets that produce lower total and low-density lipoprotein cholesterol levels”* [8]. According to the SINU position paper, most cohort studies showed that vegetarian diets had positive effects on plasma parameters. In most randomized controlled trials, vegetarian diets significantly reduced LDL-C levels [77].

### 3.5. Hypertension

According to the European Society of Hypertension (ESH), hypertension is defined as blood pressure values ≥140/90 mmHg, while according to the American College of Cardiology/American Heart Association (ACC/AHA) hypertension is defined as blood pressure values ≥130/80 mmHg [92]. Arterial hypertension is called “the silent killer”, because it can be asymptomatic unless a major vascular complication occurs. Risk factors for hypertension include—in addition to smoke, inactivity, and alcohol—also OO, T2D, and unhealthy diets. A reduction of 5 mmHg in systolic values has been associated with reductions of −7% in total mortality, −9% in cardiac mortality, and −14% in stroke mortality [93].

In the Adventist cohort, the odds of hypertension, compared to meat-eaters, were −8% in semi-vegetarians, −43% in LOVs, and −63% in VNs [94]. A pivotal intervention study performed in 1983 found that a LOV diet was able to reduce both systolic and diastolic blood pressure [95].

Meta-analyses of observational studies consistently show that a plant-based diet is associated with lower both systolic and diastolic blood pressure. In a meta-analysis of 32 observational studies, vegetarian diets were associated with lower mean systolic and diastolic blood pressure, compared to omnivorous diets [96]. For VNs, a reduction of −2.56 mmHg for systolic and −1.33 for diastolic blood pressure was reported by Benatar et al. However, a distinction between non-Asian and Asian subsamples showed a significant reduction in systolic and diastolic blood pressure only in the non-Asian population [73].

Meta-analyses of intervention trials, on the other hand, did not consistently show a significant reduction in blood pressure in vegetarians [75,85,96]. See Table 5 for details.

### 3.6. Metabolic Syndrome

Metabolic Syndrome (MetS) is not univocally defined (see NCEP, IDF, and WHO criteria [97]), but it results from the combination of several cardiometabolic factors (adiposity, blood pressure, glucose, triglyceride, and HDL-C blood levels). Its common denominator is insulin resistance. Both plant foods and a low-fat vegan diet can improve insulin sensitivity [52,98], while, on the contrary, BW and iron stores are associated with insulin resistance [99,100].

In the Adventist cohort, a cross-sectional study performed on 773 subjects found that plant-based diets (vegetarians and semi-vegetarians) were associated with a reduced risk of MetS (−56%) and the parameters composing MetS resulted more favorable in vegetarians [101]. Chiang et al. also described a reduced risk of insulin resistance (−29%) and of MetS (−57% and −54% according to the ICD criteria, in the two subgroups following a vegetarian diet for more or less than 11 years, respectively) in Asian vegetarians [102].

Picasso et al. conducted a meta-analysis on MetS [103]. Despite the variables contributing to the MetS being positively influenced by a plant-based diet, as we have described in the previous sections, the risk of MetS appeared to be non-significantly reduced in observational studies, and the association was absent in intervention studies among vegetarians. The lack of consistency of the studies on MetS, due to the different classifications applied, is confirmed also in the SINU position paper [77].

### 3.7. Vascular Disease

As a result of the above discussed risk factors (excluding cancer), atherosclerosis can develop in the body vessels and, when affecting vital organs (mainly heart and brain) it causes complications collectively referred to as “Vascular Disease” (VD), the leading cause of death in the developed countries.

Prospective studies conducted in the Oxford and Adventist cohorts consistently show protection against VD in vegetarians: respectively, −32% (IHD death and hospitalization (Oxford, [104]), and −55% (IHD mortality), −42% (VD mortality) in VN, and −23% (VD mortality) in LOV (Adventist men, [105]). A collaborative study by Key et al. on more than 76,000 subjects found a significant reduction in IHD mortality in vegetarians (−24% for vegetarians, −34% for LOVs, −26% for VNs) [106]. In two cohorts in Taiwan, vegetarians had a lower risk of total, ischemic, and hemorrhagic strokes (Cohort 1, −74% for ischemic stroke; Cohort 2, −48% for overall stroke, −59% for ischemic stroke, −66% for hemorrhagic stroke) [107].

In the unique intervention study performed by Ornish in the Lifestyle Heart Trial, CAD patients underwent coronary arteriography before and after the 5-year intervention period with a lifestyle intensive program including a vegetarian very low-fat diet (10% fat). While the control group experienced a 27.7% relative worsening in the average percent stenosis, the intervention group showed a 7.9% relative improvement, and cardiac events occurred 2.5 more frequently in the subjects who did not undergo the intervention [108].

In the various meta-analyses, incidence and mortality for IHD were consistently lower in vegetarians, compared to omnivores [65,66,109,110] (see Table 6), while no significant difference was found for stroke.

We can summarize the content of this paragraph with the words contained in the AND position paper: “Vegetarian diets are associated with a reduction in the risk of CVD. Vegetarian diets improve several modifiable heart disease risk factors, including abdominal obesity, blood pressure, serum lipid profile, and blood glucose. They also decrease markers of inflammation such as C-reactive protein, reduce oxidative stress, and protect from atherosclerotic plaque formation. Consequently, vegetarians have reduced risk of developing and dying from ischemic heart disease. Vegan diets seem to be most beneficial in improving heart disease risk factors” [8]. The SINU position paper also states: “Cohort studies showed advantages of vegetarian diets compared to non-vegetarian diets on incidence and/or mortality risk for ischemic heart disease” [77].

## 4. Conclusions

Healthy vegetarian diets are based on the consumption of unprocessed plant foods; this means that the ratio of protective to harmful compounds is higher in plant-based diets than in animal-based diets. We decided to include in this review only the pathological conditions for which there is relative consistency in the research studies. The meta-analyses we discussed have been conducted on observational and on intervention studies including adult subjects. To our knowledge, no other meta-analyses on vegetarian patterns in different life stages have been published. 

As shown by the literature in the field, vegetarians have a reduced risk of many chronic diseases, and vegetarian diets, mainly in the low-fat vegan variant, can serve as effective tool in the management of people affected by one or more chronic disease. Nevertheless, the influence of other factors on health status, such as exercise, socio-economic status, consumption of alcohol, and smoking should be considered. Although in the statistical analysis the results are adjusted for these confounding variables, their effect is not easily recognizable and can have contributed to the better health status of vegetarians.

We hope that future research will add more consistency to the remaining uncertainties that still exist, given the inherent limits of the epidemiology in nutrition.

## Figures and Tables

**Table 1:** Summary of the meta-analyses on the effect of vegetarian diets on Body Weight (BW) and Body Mass Index (BMI), compared to non-vegetarian diets (habitual or therapeutic).

	Vegetarian	LOV	VN
Observational Studies (BMI, kg/m^2^)			
Dinu, 2017 [66]		−1.49	−1.72
Benatar, 2018 [73]			−1.72 total −1.92 non-Asians −0.2 Asians ^1^
Intervention Studies (BW, kg)			
Barnard, 2015 ^2^ [74]			−4.6
Huang, 2016 ^3^ [72]	−2.02	−1.48	−2.52
Termannsen, 2022 ^2^ [75]			−7.4
Termannsen, 2022 ^3^ [75]			−2.7
Wang, 2015 ^2,3^ [76]	−2.88		

^1^ Non-significant. ^2^ Versus omnivore controls following habitual diet. ^3^ Versus omnivore controls following another specific diet for weight loss.

**Table 2:** Summary of the meta-analyses on the effect of vegetarian diets on glucose and HbA1c blood levels, compared to non-vegetarian diets.

	Vegetarian	LOV	VN
Observational Studies (Glucose Blood Levels, mmol/L)			
Dinu, 2017 [66]		−0.28	−0.35
Benatar, 2018 [73]			−0.23 total −0.39 non-Asians −0.12 Asians
Intervention Studies (HbA1c, %)			
Yokoyama, 2014 [84]	−0.39		
Viguiliouk, 2019 ^1^ [85]	−0.29		
Termannsen, 2022 ^2^ [75]			−0.18

^1^ Diabetic subjects, versus other specific diets. ^2^ Versus non-vegetarian habitual or other specific diets.

**Table 3:** Summary of the meta-analyses of observational studies on the effect of vegetarian diets on total cholesterol (TC), LDL-cholesterol (LDL-C), and HDL-cholesterol (HDL-C) blood levels, compared to non-vegetarian diets.

	TC (mg/dL)	LDL-C (mg/dL)	HDL-C (mg/dL)
Vegetarian			
Yokoyama, 2017 [88]	−29.2	−22.2	−3.6
LOV			
Dinu, 2017 [66]	−28.2	−21.3	−2.7
VN			
Dinu, 2017 [66]	−31.0	−22.3	−1.5 ^1^
Benatar, 2018 [73]		−18.9 total −23.17 non-Asians −6.18 Asians ^1^	

^1^ Non-significant.

**Table 4:** Summary of the meta-analyses of intervention studies on the effect of vegetarian diets on total cholesterol (TC), LDL-cholesterol (LDL-C) non-HDL-cholesterol (non-HDL-C), and HDL-cholesterol (HDL-C) blood levels, compared to non-vegetarian diets.

	TC (mg/dL)	LDL-C (mg/dL)	HDL-C (mg/dL)
Vegetarian			
Yokoyama, 2017 [88]	−12.5	−12.2	−3.4
Wang, 2015 [76]	−14.0	−13.0	−3.9
Viguiliouk, 2019 ^1^ [85]		−4.63 (LDL-C) −5.02 (non-HDL-C)	−1.16 ^2^
VN			
Termannsen, 2022 ^3^ [75]	−11.6	−9.3	−2.3 ^2^

^1^ Diabetic subjects, versus other specific diets. ^2^ Von-significant. ^3^ Versus non-vegetarian habitual or other specific diets.

**Table 5:** Summary of the meta-analyses of observational and intervention studies on the effect of vegetarian diets on blood pressure levels, compared to non-vegetarian diets.

	Vegetarian	VN
Observational Studies (Syst/Diast mmHg)		
Yokoyama, 2014 ^1^ [96]	−6.9/−4.7	
Benatar, 2018 [73]		−2.56/−1.33 total −5.87/−3.19 non-Asians −0.99/−0.33 Asians ^2^
Intervention Studies (Syst/Diast mmHg)		
Yokoyama, 2014 [96]	−4.8/−2.2	
Viguiliouk, 2019 ^3^ [85]	−0.1/0.53 ^2^	
Termannsen, 2022 ^1^ [75]		−1.28/−0.54 ^2^

^1^ Versus non-vegetarian habitual or other specific diets. ^2^ Non-significant. ^3^ Diabetic subjects, versus other specific diets.

**Table 6:** Summary of the meta-analyses of observational studies on the effect of vegetarian diets on vascular disease, compared to non-vegetarian diets.

	Vegetarian
Ischemic Heart Disease (IHD)	
Huang, 2012 [65]	−29% ^1^
Dinu, 2017 [66]	−25% ^1, 2^
Glenn, 2019 [109]	−22% ^2^ −28% ^1^
Dybvik, 2023 [110]	−21% ^1^
Cardiovascular Disease (CVD)	
Dybvik, 2023 [110]	−15% ^1^

^1^ Mortality. ^2^ Incidence.

## Data Availability

No new data were created or analyzed in this study. Data sharing is not applicable to this article.
